# Increased stiffness of omental arteries from late pregnant women at advanced maternal age

**DOI:** 10.1042/BSR20230819

**Published:** 2023-08-22

**Authors:** Amy L. Wooldridge, Christy Chan, Floor Spaans, Anita Quon, Craig D. Steinback, Margie H. Davenport, Sandra T. Davidge, Christy-Lynn M. Cooke

**Affiliations:** 1 Department of Obstetrics and Gynecology, Faculty of Medicine and Dentistry, University of Alberta, Edmonton, Alberta, Canada; 2 Women and Children’s Health Research Institute, University of Alberta, Edmonton, Alberta, Canada; 3 Faculty of Kinesiology, Sport, and Recreation, University of Alberta, Edmonton, Alberta, Canada; 4 Department of Physiology, Faculty of Medicine and Dentistry, University of Alberta, Edmonton, Alberta, Canada

**Keywords:** advanced maternal age, elastin, maternal outcomes, omental artery, pregnancy, vascular structure

## Abstract

Advanced maternal age (≥35 years) is a risk factor for poor pregnancy outcomes. Pregnancy requires extensive maternal vascular adaptations, and with age, our blood vessels become stiffer and change in structure (collagen and elastin). However, the effect of advanced maternal age on the structure of human resistance arteries during pregnancy is unknown. As omental resistance arteries contribute to blood pressure regulation, assessing their structure in pregnancy may inform on the causal mechanisms underlying pregnancy complications in women of advanced maternal age. Omental fat biopsies were obtained from younger (<35 years) or advanced maternal age (≥35 years) women during caesarean delivery (*n* = 7–9/group). Arteries (200–300 µm) were isolated and passive mechanical properties (circumferential stress and strain) assessed with pressure myography. Collagen (Masson’s Trichrome) and elastin (Verhoff) were visualized histologically and % positively-stained area was assessed. Median maternal age was 32 years (range 25–34) for younger, and 38 years (range 35–42) for women of advanced maternal age. Circumferential strain was lower in arteries from advanced maternal age versus younger women but circumferential stress was not different. Omental artery collagen levels were similar, while elastin levels were lower with advanced maternal age versus younger pregnancies. The collagen:elastin ratio was greater in arteries from advanced maternal age versus younger women. In conclusion, omental arteries from women of advanced maternal age were less compliant with less elastin compared with arteries of younger controls, which may affect how vascular stressors are tolerated during pregnancy. Understanding how vascular aging affects pregnancy adaptations may contribute to better pregnancy outcomes.

## Introduction

An increasing number of women are having pregnancies at an advanced maternal age (≥35 years). In Canada, over a quarter of live births are now from pregnancies at advanced maternal age [1]. However, advanced maternal age is a well-known risk factor for not only fetal chromosomal abnormalities, but also poor pregnancy outcomes such as preeclampsia and intrauterine growth restriction [2]. The latter increased risk for adverse outcomes may be due to impaired maternal vascular adaptations to pregnancy. Achieving a healthy pregnancy requires extensive maternal hemodynamic adaptations, including expansion of blood volume, a ∼50% increase in cardiac output, and an overall decrease in peripheral vascular resistance [3]. It is important to study cardiovascular changes with advanced maternal age, as it may relate to the increased risk of adverse pregnancy outcomes in this population.

Arterial compliance and stiffness are determined by the structural composition of the vessel walls. Collagen and elastin protein fibers are extracellular structural fibers found in blood vessels, and they are the main determinants of passive mechanical properties of arteries (reviewed in [4]). Collagen is important for protection against vessel rupture and limiting arterial stretch, and elastin enables continuity of blood flow by allowing arteries to distend in response to pressure, thereby reducing fluctuations in local blood pressure in response to ventricular contractions [4–6]. The splanchnic circulation is a major contributor to peripheral vascular resistance, and arterial function in this region undergoes adaptations during pregnancy (reviewed in [7]) to allow consistent perfusion pressure despite increased blood flow generated by greater blood volume and cardiac output. Arterial tone is determined by a combination of active function and passive structure.

Aging is associated with vascular stiffness; however, these findings come from non-pregnant populations and animals that are well over the age of reproductive senescence. Previous studies by our lab demonstrated that advanced maternal age in rats impairs both vascular function and structure. Uterine arteries from aged pregnant rats have altered circumferential stress–strain relationships, including less strain, compared with younger pregnant controls [8]. Decreased strain values, with a leftward shift of the passive circumferential stress–strain curve, indicate reduced compliance [9]. These vascular changes were accompanied by reduced litter size, lowered fetal placental weight ratio, and increased fetal resorptions [8,10], linking poor vascular adaptations during pregnancy with poor pregnancy outcomes. Whether there are any potential effects on vascular stiffness in an older yet still fertile population of ‘advanced’ maternal aged women during their pregnancies is not known. It may be that there are subtle age-related changes in the vascular system that may contribute to the increased risk of adverse pregnancy outcomes in this population.

While Doppler imaging has also confirmed increased uterine artery impedance in women of advanced maternal age [11], the interaction between maternal age and pregnancy and how it affects the human systemic resistance arteries, which regulate blood pressure, remain poorly understood [12]. Age-related changes do not occur uniformly across the vascular system [13], so considering the large contribution of omental resistance arteries to systemic blood pressure, it is important to assess the effects of maternal age on these arteries as well. Omental arteries, which are located in sheets of adipose tissue lining the abdomen, can be safely obtained from women during Caesarean section deliveries. The omental fat is highly vascularized, contributing substantially to splanchnic blood flow [14]. Therefore, the omental arteries are accessible vessels for *ex vivo* study of the difference in pregnancy adaptations with advanced maternal age.

### Hypothesis

To the best of our knowledge, the current study represents the first to directly assess the impact of advanced maternal age on the structure of human omental resistance arteries. We hypothesized that women of advanced maternal age would have less compliant omental resistance arteries with greater stiffness, and increased collagen and decreased elastin content, informing on potential causal mechanisms for the increased risk of pregnancy complications in women of advanced maternal age.

## Methods

### Tissue collection

This study was approved by the University of Alberta Research Ethics Board (Pro00000944) and all patients provided informed, written study consent prior to their caesarean surgery (see Ethics Statement for full details). A small (1–2 cm^2^) piece of omental tissue was collected from younger women (18-34 years) and women of advanced maternal age (≥35 years) undergoing scheduled caesarean deliveries of singletons at the Lois Hole Hospital for Women at the Royal Alexandra Hospital in Edmonton, Alberta, between May 2022 and January 2023. Women with significant comorbidities (i.e. type II diabetes, pre-existing cardiovascular disease or preeclampsia) were excluded. Two of the ten women in the advanced maternal age group had *in vitro* fertilization-assisted pregnancies, of which one was noted to have used a donor egg. None of the eleven women in the younger group were noted as having had *vitro* fertilization-assisted pregnancies. Data on maternal age, race, gestational age, pre-pregnant BMI, obstetrical history, blood pressure, and infant outcomes such as birth weight, sex and Apgar score were obtained from medical records. Blood pressure data represents the pre-operative blood pressure at admission on the day of Caesarean delivery.

Upon removal, the omental biopsy was immediately placed into ice-cold HEPES-buffered physiological saline solution (PSS; composition [in mM]: 142 NaCl, 10 HEPES, 4.7 KCl, 1.17 MgSO_4_, 1.18 KH_2_PO_4_, 1.6 CaCl_2_, 5.5 glucose, and pH 7.4) and transported to the laboratory within the Heritage Medical Research Centre in Edmonton for processing. Omental arteries (diameter 200–300 µm) were immediately isolated for assessment of *ex vivo* vascular mechanical properties. Additional omental arteries were embedded in Optimal Cutting Temperature compound and stored at −80°C for later histological analysis.

### Pressure myography

Mechanical properties (circumferential stress and strain) of isolated omental arteries were assessed using pressure myography. Arteries were mounted on two glass cannulas in a pressure myograph system (Living Systems, Burlington, NJ, U.S.A.) filled with calcium-free solution (in mmol/L: 142 NaCl, 10 HEPES, 4.7 KCl, 1.17 MgSO_4_, 1.18 KH_2_PO_4_, 2 EGTA, and pH 7.4) at 37°C. Prevention of exposure to calcium ions (to eliminate ‘active’ constriction and myogenic tone) was confirmed through the addition of ethylene glycol tetraacetic acid (EGTA) to chelate the calcium ions and intracellular calcium stores were depleted by adding papaverine (0.1 µmol/L; P3510, Sigma) to the bath. Intravascular pressure was monitored and adjusted using a pressure servo control PS/200 peristaltic pump and a perfusion pressure monitor. Intraluminal and wall diameters were measured using a Lasico digital filar eyepiece (model 1602E-10) and processor attached to a stereo microscope (Olympus SZH10). Following a 20 min equilibration at 60 mmHg, passive characteristics were assessed using a pressure curve. The intraluminal diameters of arteries were measured in 20 mmHg steps across a pressure range of 4–160 mmHg, and the vessels were left at each pressure for at least one minute prior to measurement. Circumferential wall stress and strain were analyzed as described previously [10,15] and differences assessed by comparing area under the curve (AUC).

### Histological analysis of collagen and elastin

OCT-embedded arteries (*n* = 7–9/group) were sectioned at 8 µm thickness and stained for either collagen or elastin. Collagen was visualized using Masson’s Trichrome staining on formalin-fixed sections using a kit as per manufacturer instructions (cat#HT15-1KT, with the required additional Bouin’s solution cat#HT10132 and Weigart’s iron hematoxylin solution set cat#HT1079, Sigma Aldrich). Elastin was visualized using Verhoff’s staining [16] of tissue sections fixed in Baker’s solution (0.75 g CaCl_2_ and 7.5 ml of 37% formaldehyde with deionized water added to make a total of 250 ml) for 10 min. Slides were then exposed for 15 min to Verhoff solution (0.25 g powdered hematoxylin [cat#47223, Alfa Aesar] in 5 ml of absolute ethanol, mixed well with 2 ml of 10% ferric chloride [cat#217091000, ThermoFisher Scientific] in deionized water before the addition of 2 ml of Lugol’s iodine solution [0598-05, Medisca, Richmond, BC, Canada]) before they were rinsed with water. The tissue sections were exposed to a solution of 1% ferric chloride in water until elastin structures were clearly visible, then rinsed with water and counterstained using Fast Green (cat#A16520-06, Alfa Aesar, Tweksbury, MA, U.S.A.). After staining, tissue sections were dehydrated and sealed with DPX solution using a cover slip. Slides were imaged at 40× magnification using bright field microscopy (EVOS XL Core Imaging System, ThermoFisher Scientific, Waltham, MA, U.S.A.). Collagen and elastin within the arterial tunica media were analyzed in a blinded manner using ImageJ software (NIH), employing color deconvolution and percent positive area tools.

### Statistical analyses

Data were analysed using GraphPad Prism software (v9.5.1, San Diego, U.S.A.). Patient demographics were assessed by *t*-test or χ^2^-test, as appropriate. The effect of age on outcomes were assessed using a *t*-test. Grubb’s test was used to exclude significant outliers in the data sets. Data are presented as mean ± SEM. *P*<0.05 was considered statistically significant.

## Results

### Maternal demographics

Patient demographics are detailed in Table 1. Besides maternal age, infant sex also differed, as the pregnancies included in the advanced maternal age group had a greater proportion of female infants (70%), whereas the pregnancies included in the younger group had a greater proportion of male infants (82%, *P*=0.0166). Systolic blood pressure within 24 h of delivery tended to be lower in women of advanced maternal age than in younger women (*P*=0.0605). No other patient demographic data differed between younger and advanced maternal age groups (see Table 1).

**Table 1:** Demographics and clinical parameters of women included in the study

	Younger (*n*=11)	Advanced maternal age (*n*=10)	*P*-value
**Age (years)**, median (IQR)	32.0 (29.0–32.0)	38.0 (36.0–40.5)	**<0.0001^‡^**
**Ethnic origin**, *n* (%)			ns*
Caucasian	8 (73%)	5 (50%)	
Hispanic	1 (9%)		
Asian		1 (10%)	
Indian		2 (20%)	
Filipino		1 (10%)	
Black African	1 (9%)	1 (10%)	
Egyptian	1 (9%)		
**Gravidity**, median (IQR)	3.00 (2.00–4.00)	2.50 (1.75–4.25)	ns^†^
**Abortions**, median (IQR)	1.00 (0.00–2.00)	0.00 (0.00–1.25)	ns^†^
**Smoked during pregnancy**, number (% of group)	0 (0%)	1 (10%), cannabis	ns*
**Pre-pregnancy BMI (kg/m^2^)**, median (IQR)	24.35 (23.03–28.63)	26.30 (22.95–33.35)	ns†
**Systolic blood pressure (mmHg)**, mean ± SEM	121.5 ± 2.7	114.3 ± 2.2	**=0.0605^‡^**
**Diastolic blood pressure (mmHg)**, mean ± SEM	75.6± 2.6	69.8 ± 2.0	ns^‡^
**Pulse pressure (mmHg)**, mean ± SEM	43.4 ± 2.0	44.7 ± 1.5	ns^‡^
**Gestational age (days)**, mean ± SEM	271.4 ± 0.9	273.1 ± 1.1	ns^‡^
**Infant birth weight (g)**, mean ± SEM	3525 ± 129	3391 ± 125	ns^‡^
**Infant sex**, males number (% of group)	9 (82%)	3 (30%)	= 0.0166*
**Infant Apgar (1 min)**, median (IQR)	9.0 (9.0–9.0)	8.5 (6.75–9.0)	ns^†^
**Infant Apgar (5 min)**, median (IQR)	9.0 (9.0–9.0)	9.0 (7.75–9.0)	ns^†^

*χ^2^ test, †Mann–Whitney *U-*test, ‡*t*-test.

BMI and blood pressure data was not available for one younger patient and one patient of advanced maternal age. Gravidity: number of pregnancies; abortions: number of losses before 20 weeks. BMI, body mass index; IQR, interquartile range; ns, not significant; SEM, standard error of the mean.

### Omental arteries of pregnant women of advanced maternal age were stiffer than omental arteries of younger pregnant women

**Figure 1:**
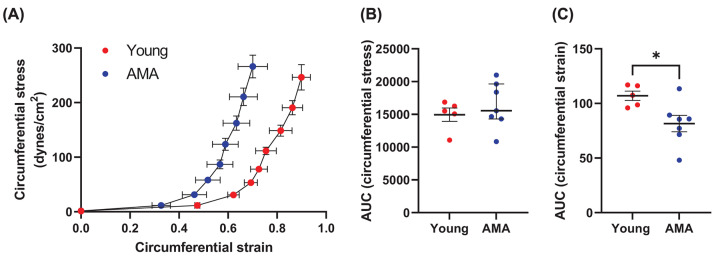
Circumferential stress-strain properties (**A**) Circumferential stress-strain relation of omental resistance arteries from pregnant younger (young, <35 years) and advanced maternal age (AMA, ≥35 years) women. (**B**) Area under the curve (AUC) for circumferential stress. (**C**) AUC for circumferential strain; *n* = 5–7 women/group, *P<0.05.

Omental resistance arteries from pregnant women of advanced maternal age had less circumferential strain (AUC, *P*=0.0250, Figure 1A,C) than those from pregnant younger women, with no difference in circumferential stress (Figure 1A,B). A leftward shift in the circumferential stress–strain curve for arteries from pregnant women of advanced maternal age relative to the arteries from younger pregnant women (Figure 1A) represents an increase in stiffness.

### Omental arteries of pregnant women of advanced maternal age had less elastin and a greater collagen:elastin ratio than the omental arteries of younger pregnant women

**Figure 2:**
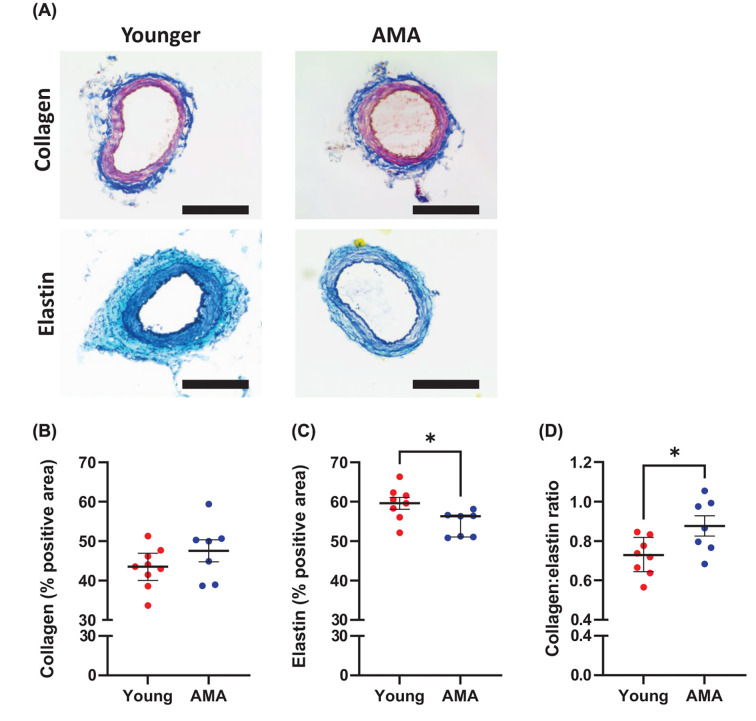
Histological structure (**A**) Representative histology images of omental resistance arteries from pregnant younger (young, <35 years) and advanced maternal age (AMA, ≥35 years) women, stained for collagen and elastin fibers using Masson’s Trichrome (blue = collagen, red = smooth muscle) and Verhoef’s stains (black = elastin, green = background), respectively. Scale bar: 200 μm. (**B**) Percent positive area of collagen in the tunica media. (**C**) Percent positive area of elastin in the tunica media. (**D**) Ratio of the percent positive areas of collagen:elastin. *n* = 7–9 women/group, *P<0.05.

Omental resistance arteries from pregnant women of advanced maternal age had a lower percent positive area of elastin (*P*=0.0195, Figure 2A,C) than those from pregnant young women, with no difference in percent positive area of collagen (Figure 2A,B) in the tunica media. This resulted in arteries from pregnant women of advanced maternal age having a greater collagen:elastin ratio than those from pregnant young women (*P*=0.0243, Figure 2D).

## Discussion

The current study is the first to assess the effects of advanced maternal age on the structural characteristics of omental resistance arteries from otherwise healthy pregnant women. Using *ex vivo* assessment methods, we have directly shown that advanced maternal age affects the structure of omental arteries in pregnant women resulting in reduced compliance of these systemic vessels. Indeed, omental arteries from women of advanced maternal age were stiffer (less compliant) and contained less elastin, which resulted in a greater collagen:elastin ratio compared with omental resistance arteries from younger pregnant women. Overall, we identified age-related structural changes in the systemic vasculature of pregnant women that may contribute to the greater risk of poor pregnancy outcomes in women of advanced maternal age.

We showed that circumferential strain (deformation due to stress) is lower in omental resistance arteries from women of advanced maternal age than in arteries from younger pregnant women. This represents a reduced maximum capacity for blood carrying capacity and potentially less ability to compensate for impaired vascular function when needed. Previously, using a pregnant rat model for advanced maternal age, we observed reduced circumferential strain in the uterine arteries of aged pregnant (vs. younger pregnant) rats at gestational day 20 (term = 22) [8]. In the rat model, we did not observe a difference in collagen:elastin ratio in arteries from pregnant rats of different age groups; however, during the present study, we observed that arteries from women of advanced maternal age had a greater collagen:elastin ratio compared with arteries from younger women. In another study using the same rat model, however, we observed no age-related differences in the circumferential stress and strain of systemic mesenteric arteries [10]. In the current study, we were not able to include non-pregnant control groups due to limitations in our ability to obtain surgical samples from patients before or after pregnancy, so we are unable to confirm the extent to which age-related structural differences occurred prior to or during pregnancy. However, our data clearly and directly shows that structural and compliance differences exist in arteries from pregnant women of advanced maternal age compared with pregnant women of younger age.

Pregnant preeclamptic women have been reported to have a greater augmentation index (a proxy measure of left ventricular systolic loading) [17,18], and a greater pulse wave reflection (a proxy measure of arterial stiffness) according to measurements taken of the aorta [17], carotid-radial and carotid-femoral [18] vasculature. A study of omental resistance arteries from pregnant women with preeclampsia, intrauterine growth restriction or normal pregnancies found no difference in lumen diameter, wall thickness, wall:lumen ratio, distensibility or circumferential stress–strain relationship, with no difference in histological measurements [19]. However, none of the aforementioned studies addressed maternal age. In a study of 627 uncomplicated singleton pregnancies between 10 and 40 weeks of gestation, cardiac output decreased from 25 until 32 years of age, with an increase in systemic vascular resistance over the same time period (ultrasound probe measurement of transaortic blood flow) [20]. Collectively, these data suggest that there are subclinical effects of increasing age that in the presence of secondary vascular stressors, at least, may contribute to the greater risk for pregnancy complications in women of advanced maternal age. Reduced arterial compliance may reduce the extent to which vascular function can compensate for vascular stressors and may contribute to poor pregnancy outcomes. Indeed, greater systemic vascular resistance index in the third trimester of pregnancy was found to be associated with a lower neonatal weight percentile within a cohort of advanced maternal age women, but not within younger controls (younger, *r* = −0.10, *P*=0.060; advanced maternal age, *r* = −0.062, *P*=0.002) and this association in women of advanced maternal age persisted even after the removal of pregnancies affected by hypertensive disorders from the analysis [21]. Therefore, despite not including women with hypertensive disorders in the current study, the presence of increased stiffness within the vasculature (omental resistance arteries) from women of advanced maternal age may well represent a contributing factor to poor pregnancy outcomes in women of this age group.

The demographics within our study participants were generally similar between groups. The pregnancies included within our advanced maternal age group had a greater proportion of female infants (70%), whereas the pregnancies included within our younger group had a greater proportion of male infants (82%). This may have resulted in a somewhat biased sample of advanced maternal age women, as pregnancies with male fetuses (particularly with advanced maternal age) tend to have more complications [22], or potentially different complications than pregnancies with female fetuses [23]. Additionally, our focused inclusion of scheduled Caesarean deliveries would have prevented the inclusion of more complicated pregnancies requiring emergency delivery within our study. As these factors may have resulted in a stronger vascular phenotype within our advanced maternal age group, their reduced impact on our study further supports that advanced maternal age itself is having a large impact on the maternal systemic vasculature. We also observed within our patients that systolic blood pressure taken on the morning of hospital admission tended to be lower within women of advanced maternal age than in younger women, whereas diastolic blood pressure did not differ between age groups. While our study sample was small with *n*=21 women in total, a larger prospective study of 8,623 women previously reported that maternal age was positively associated with diastolic blood pressure in the third trimester (additional 0.5 mmHg per 10 years of maternal age), with no difference in systolic blood pressure [24]. It is possible that the increased vascular stiffness may result in an inability to compensate for secondary stressors, but this remains an area for future study.

To investigate the mechanisms behind reduced circumferential strain with aging in pregnant women, we histologically assessed collagen and elastin content of the tunica media from omental resistance arteries, as they affect vascular compliance and stiffness. Omental arteries from women of advanced maternal age contained less elastin content, which resulted in a greater collagen:elastin ratio, compared with omental arteries from younger women. These structural differences corroborate our pressure myography findings, as elastin confers vascular elasticity when permitted by other vascular components. In the current study, we observed less elastin in omental resistance arteries from older compared with younger women, which may be considered a very early sign of vascular aging, at least in that the capacity to cope with adaptations to pregnancy may already be compromised in women at or above 35 years of age. As age-related degradation of elastin does not occur significantly until 60–70 years of age in humans [25], our findings may represent lower turnover of elastin in women of advanced maternal age. In aging rats, there is a decrease in carotid artery tunica media content of elastin and its synthesis enzyme lysyl oxidase, together with an increase in the elastin-degrading enzyme matrix metalloproteinase-2 [26]. Future studies should focus on the mechanisms behind the structural vascular changes identified in the present study.

Advanced maternal age affects an increasing proportion of pregnancies and is associated with pregnancy complications. Maternal vascular adaptations during pregnancy are important to achieve a healthy pregnancy. Using *ex vivo* analyses of systemic omental resistance arteries, we have shown that advanced maternal age is associated with increased vascular stiffness and reduced compliancy, due to a lower elastin content and greater collagen:elastin ratio. Thus, advanced maternal age may limit the ability of these systemic blood vessels to compensate for vascular stressors.

## Future directions

As omental arteries contribute to the regulation of blood pressure, this may be a potential mechanism by which cardiovascular-related pregnancy complications such as preeclampsia occur at a greater rate in pregnant women of advanced maternal age. Yet, the mechanisms behind the age-related differences in the structure of systemic arteries remain uncertain. Future studies should aim to identify potential targets, which may involve matrix metalloproteinases and their regulating factors. Additional studies should also confirm whether the interaction between systemic resistance artery stiffness and age relates to poor pregnancy outcomes. Although the present study was limited to the assessment of omental resistance arteries, there may also be other vascular beds (such as the myometrial vascular bed) with greater resistance artery stiffness—which provide another area of interest for future research.

## Data Availability

The data supporting this study are available on request from the corresponding author.

## Abbreviations

AUC: area under the curve
BMI: body mass index
IQR: interquartile range
PSS: physiological saline solution
